# Effects of Biological Characteristics and Environmental Factors on Swimming Performance of Endemic Fish in Southwest China

**DOI:** 10.3390/ani15121819

**Published:** 2025-06-19

**Authors:** Jianing Rao, Zhiguang Zhang, Yuanming Wang, Qi Wei, Guoqing Chen, Xintong Li, Ruifeng Liang, Kefeng Li

**Affiliations:** 1 State Key Laboratory of Hydraulics and Mountain River Engineering, Sichuan University, Chengdu 610065, China; 2 Beijing Engineering Corporation Limited, Power Construction Corporation of China, Beijing 100024, China; 3 Sichuan Water Development Investigation, Design & Research Co., Ltd., Chengdu 610072, China

**Keywords:** biological characteristics, endemic fish, environmental factors, swimming performance, driving factors

## Abstract

Swimming is crucial to fish survival and reproduction, and is usually influenced by species, morphology, habitat environment and physiological condition. Understanding the changes in fish swimming ability and identifying the driving factors behind these changes is vital. In this study, seven endemic fish species from the Qingshui River, an important tributary of the upper Pearl River in Southwest China, were used to explore the effects of biological and environmental factors on fish swimming ability. The results indicated that the body length parameters had a minimal effect on the induced swimming speed (U_ind_) but played a positive role in critical and burst swimming ability (U_crit_ and U_burst_). Weight (W) and W/SL (the ratio of weight to standard body length) correlated more highly with swimming ability than body length parameters. Fish preferring the lotic environment exhibited higher U_crit_ and U_burst_, and fish with a streamlined morphology had a smaller U_ind_. The U_ind_, U_ind_/SL, U_ind_/W and U_ind_/(W/SL) of *Discogobio yunnanensis* (Regan, 1907) and *Pseudocrossocheilus tridentis* (Cui & Chu, 1986) were significantly higher during the spawning period. *Acrossocheilus yunnanensis* (Regan, 1904) performed better during the spawning period in U_crit_ and U_crit_/SL. The U_burst_ of fish during the spawning period was smaller than that during the non-spawning period. Generally, the species difference had the greatest contribution to the swimming ability difference, followed by environmental conditions and fish morphology.

## Simple Summary

Fish swimming is crucial to their survival and reproduction, and is usually influenced by species, morphology, habitat environment and physiological condition. Understanding the changes in fish swimming ability and identifying the driving factors behind these changes are of pressing importance. Our findings showed that body length parameters had a minimal effect on the induced swimming speed, but played a positive role in critical and burst swimming ability. Weight parameters were more highly correlated with swimming ability than body length parameters. Fish preferring the lotic environment exhibited higher U_crit_ and U_burst_, and fish with a streamlined morphology had smaller U_ind_. The burst swimming speed of fish during the spawning period was slower than that during the non-spawning period. Generally, the swimming ability was mainly influenced by the species identity, followed by environmental conditions and morphological factors. The findings of this study are of great significance to the conservation of rare and endemic fish in mountainous rivers, and could provide a theoretical basis and data support for the design of river restoration measures.

## 1. Introduction

Fish swimming is essential for their survival and reproduction, and is related to the achievement of behaviors such as schooling, migration, reproduction, foraging and predator avoidance [1,2]. The strength of fish’s swimming ability directly influences the sustainability of fish populations and is of great ecological importance [3]. Fish’s anaerobic exercise relies on the size of their white muscle mass and aerobic exercise is supported by red muscle tissue [4,5,6], leading to the introduction of three important metrics: induced swimming speed (U_ind_), critical swimming speed (U_crit_) and burst swimming speed (U_burst_) [7]. These swimming metrics help to distinguish fish swimming speeds intuitively, and can be used to evaluate the fish migratory movements.

Differences in habitat environment, morphology and life periods have led to the evolution of fish with different swimming performances [8]. Over a long evolutionary history, different fish species have developed corresponding swimming abilities adapted to their living environment. For example, fish living in the lotic environment showed a preference for high flow velocities, while those in the lentic environment were weaker swimmers [9]. Different morphologies shape the different swimming capabilities of fish. The spindle-shaped body makes *Cottus bairdii* (Girard, 1850) and *Rhinichthys cataractae* (Valenciennes, 1842) good swimmers for long-distance migration [10,11], while lateral flattened fish were found to be more susceptible to the disturbance caused by water currents than spindle-shape ones [12]. Reproduction is a critical period in the life cycle of fish when the energy requirement of fish is at its highest [13]. During reproduction, potamodromous freshwater fish allocate energy to gamete production, potentially reducing energy for locomotion, as predicted by the trade-offs from biological evolution theory [14]. Swimming performance varies by species and life periods [15,16]. Due to spawning, fish usually optimize their allocation of energy by subduing the locomotor ability to compensate for reproduction. However, the differences in fish swimming ability between spawning and non-spawning periods has received less attention.

Currently, the construction and operation of massive hydropower projects all over the world have blocked fish migration in rivers [17,18,19,20], further affecting the survival of fish. These hydropower projects have also significantly altered the hydrodynamic characteristics of the rivers, leading to changes in the flow field suitable for fish spawning [21]. The disappearance of some shoals, deep pools and rapids simplifies the complex river system, and what is worse, the environment for fish reproduction may also be destroyed [22]. In recent years, fish passage facilities have constantly emerged to restore the deteriorated connectivity of rivers [23]. The successful design of fish passage facilities not only depended on the hydrodynamic characteristics, but was also closely related to the biological characteristics of the target fish species which were in need of migration. Fish swimming performance is the key metric to guarantee passing efficiency. For example, 52% of *Hybognathus amarus* passed over the dual vertical-slot fishway in Grand River with a less turbulent flow of 78 cm/s, while the passage rate decreased to 8% at a faster and more turbulent flow of 87 cm/s, which exceeded the U_crit_ of *Hybognathus amarus* [24].

There are abundant fish resources and a wide range of endemic fish species in Southwest China [25,26]. However, most of the current studies on fish swimming ability are species-specific, and have difficulty in helping to comprehensively understand the key factors driving fish swimming ability. The hydropower resources in China are mainly concentrated in the Southwest region [27]. Under the impetus of the western development and West-to-East Power Transmission policies enacted recently, the construction of hydropower projects in major basins of the Southwest has been accelerated to an unprecedented degree [27,28], causing the ecological destruction of the river ecosystem to be increasingly prominent. Relevant environmental impact assessments (EIAs) for hydropower projects require the deployment of fish passages, fish ramps, or fish lifts to protect fish genetic integrity [29,30]. Fish swimming speeds are vital metrics to evaluate the operational efficiency of fish passage facilities and exhibit distinct preferences to water depth, water velocity and substrates [31]. Fish with a streamlined morphology usually inhabit high-velocity environments over gravel substrates, while fish with a dorsoventrally flattened morphology always occupy deeper positions with lower velocities and silt–sand substrates [32,33]. However, relevant studies on fish swimming performance were insufficient to support the increasingly strict requirements of river connectivity restoration. It is particularly urgent at this stage to reveal the patterns in the variation of fish swimming ability and explore the driving factors behind these changes. This study hypothesizes that the biological characteristics of fish and environmental conditions mainly impact fish swimming performance. Seven endemic fish species from the Qingshui River, an important tributary of the upper Pearl River in Southwest China, were taken as control species to explore the effects of biological characteristics and environmental factors on fish swimming ability. The findings of this study are vital to the conservation of rare and endemic fish in mountainous rivers, and could provide a theoretical basis and data support for the design of river restoration measures.

## 2. Materials and Methods

### 2.1. Study Area and Experimental Fish

The Qingshui River is an important tributary of the upper Pearl River in Southwest China (Wenshan City, Yunnan Province), with a total length of 134 km. A large variation in the water level leads to complex habitats, cultivating plenty of endemic fish species. Among the endemic fish in the Qingshui River basin, *Acrossocheilus yunnanensis* (Regan, 1904), *Abbottina rivularis* (Basilewsky, 1855) and *Onychostoma elongatum* (Pellegrin & Chevey, 1934) have a short-distance migration requirement during their spawning period. The Qingshui River basin is endowed with abundant water energy storage and large water-level differences. Several hydropower projects have been developed in the Qingshui River basin, adversely affecting the short-distance migration of fish and blocking the exchange of fish genes upstream and downstream of dams.

**Figure 1:**
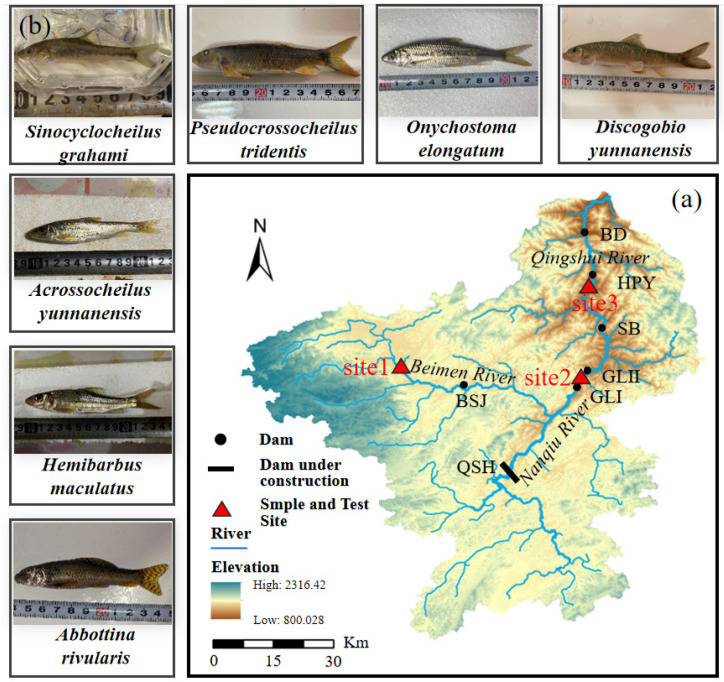
The experimental fish and their sample sites in this study. (**a**) indicates the positional relationship between the Qingshui River basin and the test sites in this study, and (**b**) shows the seven experimental fish. Qingshuihe (QSH), Bisongjiu (BSJ), Gelei I (GLI), Gelei II (GLII), Shibie (SB), Houpayan (HPY) and Bada (BD) in this figure represent the different constructed hydropower plants in this area.

The spawning period of fish in the Qingshui River Basin lasts from March to July, and fish collections were conducted in April (the spawning period) and October (the non-spawning period) in this study. Seven endemic fish species (*Pseudocrossocheilus tridentis* (Cui & Chu, 1986), *Acrossocheilus yunnanensis*, *Discogobio yunnanensis* (Regan, 1907), *Abbottina rivularis*, *Sinocyclocheilus grahami* (Regan, 1904), *Hemibarbus maculatus* (Bleeker, 1871), *Onychostoma elongatum*) were obtained in October 2020 and April 2021, respectively, at three typical sections of the Qingshui River basin (site 1: E104.28°, N24.05°; site 2: E104.52°, N24.08°; site 3: E104.52°, N24.23°; Figure 1). Obtained fish were temporarily kept in keeping tanks with natural river water and enriched oxygen for 48 h to eliminate the stress reaction of the fish transportation [34]. The seven endemic fish species were selected based on their ecological significance in the Qingshui River basin, their short-distance migration requirements during spawning and their susceptibility to hydropower-induced habitat fragmentation, making them representative objects for studying swimming performance in this basin.

After the swimming speed test finished, fish morphology parameters, including fish standard body length (SL), fish fork length (FL), fish total length (TL) and fish weight (W), were measured using digital calipers (±0.1 mm) and an electronic scale (±0.1 g), following standardized protocols [35]. SL, FL and TL were measured from the tip of the snout to the posterior end of the vertebral column (caudal peduncle), the center of the caudal fin fork and the distal tip of the longest caudal fin ray, respectively. Environmental factors, including dissolved oxygen (DO) and the temperature (T) of the river water, were measured by the dissolved oxygen portable meter (YSI Pro20).

### 2.2. Fish Swimming Ability Test

U_ind_, U_crit_ and U_burst_ are three important metrics frequently used to evaluate fish swimming ability. U_ind_ refers to the minimum speed at which a fish can discern the flow direction of water. U_crit_ is the maximum aerobic speed of a fish, which can be used to determine the maximum oxygen consumption capacity of a fish during the swimming process. U_burst_ is the transient and rapid swimming speed where fish are in a state of anaerobic respiration and lasts for less than 20 s [8,36].

**Figure 2:**
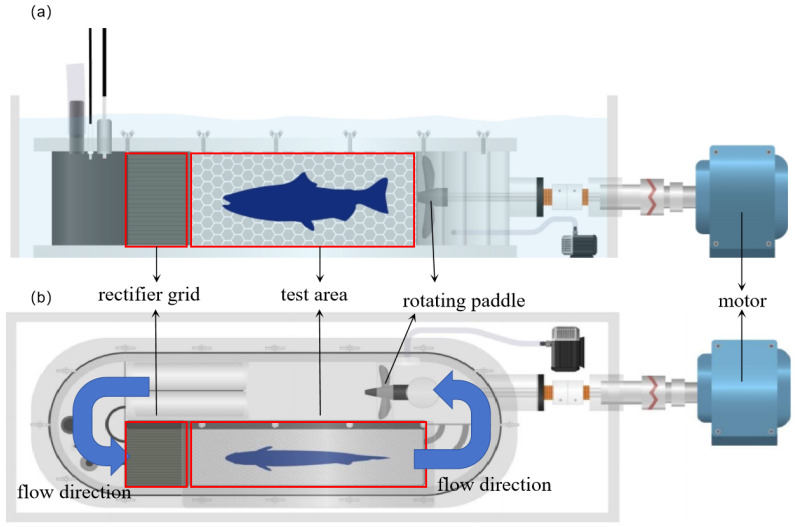
The swimming ability test system. (**a**) and (**b**), respectively, represent the front view and the top view of the device.

The swimming ability test system (SY10800, Danish Loligo Company, Viborg, Denmark), with a volume of 30 L for the sealing area and a volume of 9 L for the test chamber, was adopted to carry out the fish swimming ability test. The motor drove the rotating paddle to generate the flow velocity from 5 to 180 cm/s. A linear relationship between the propeller rotation speed and the flow velocity was built and the desired flow velocity could be obtained by controlling the rotating frequency. The front rectifier grid was provided at the inlet of the test area to stabilize the flow pattern and ensure a uniformly distributed flow velocity (Figure 2). At the beginning of the swimming ability test, fish were acclimated in the test chamber for 20 min with a velocity of 5 cm/s to eliminate the stress of the transfer process [37,38]. The increasing flow velocity method was used to test U_ind_, U_crit_ and U_burst_ in this study.

The swimming speeds were tested after fish acclimation. In terms of the fish U_ind_ test, the flow velocity was increased by 5 cm/s every 1 min from the acclimated velocity. The movement of the fish was observed until the fish exhibited rheotaxis and turned its direction to swim against the current. The flow velocity at this time was taken as the U_ind_ of the experimental fish. The calculation formula for U_ind_ is as follows [35]:

(1) $$U_{ind}=U_{p}+(t/\Delta t)\Delta U$$

where $U_{p}$ is the velocity when the fish exhibit rheotaxis, ΔU is the flow velocity increment, t is the time to start reversing course and Δt is the interval of each increase. For the U_ind_ test, 5 cm/s was taken as $U_{p}$ and 1 min was taken as Δt.

For the U_crit_ test, the flow velocity was increased by 5 cm/s every 20 min from the acclimated velocity. The movement of the fish was observed until the fish was exhausted and leaned against the downstream wire mesh for more than 2 min, when the flow velocity was taken as the U_crit_ of the experimental fish. The calculation formula for U_crit_ is as follows [39]:

(2) $$U_{crit}=U_{p}+(t/\Delta t)\Delta U$$

where $U_{p}$ is the velocity when the fish is exhausted, ΔU is the flow velocity increment, t is the time from the last increase to the fish exhaustion and Δt is the interval of each increase. For the U_crit_ test, 5 cm/s was taken as $U_{p}$ and 20 min was taken as Δt.

U_burst_ has a similar test methodology to U_crit_, except that the interval of each increase is changed to 1 min. The calculation formula for U_burst_ is as follows [35]:

(3) $$U_{burst}=U_{p}+(t/\Delta t)\Delta U$$

To eliminate the effect of body length and weight on the swimming ability, the absolute swimming metrics (U_ind_, U_crit_ and U_burst_) were divided by SL, W and W/SL, respectively, to obtain relative swimming metrics, including U_ind_/SL, U_ind_/W, U_ind_/(W/SL), U_crit_/SL, U_crit_/W, U_crit_/(W/SL), U_burst_/SL, U_burst_/W and U_burst_/(W/SL).

### 2.3. Data Analysis

Bivariate correlation was used to test the relationship between the three swimming speeds and the morphology parameters of the experimental fish, with a significance level of *p* < 0.05. The linear or power regression was applied for swimming speeds and the morphology parameters where significant correlation existed. The mantel test is a non-parametric statistical method used to evaluate the correlation between two matrices. It was used to evaluate whether fish swimming speeds had correlations with the biological characteristics (species, SL, FL, TL, W and W/SL) and environmental factors (the life periods, sample sites, water temperature and DO). We used Rstudio to implement random forest (RF) coding to rank the importance of the factors affecting fish swimming speeds. The independent samples t test was used to analyze the difference in each morphological parameter and swimming speed between the two periods for each fish species. For datasets meeting normality assumptions, one-way ANOVA was employed to test interspecies differences in swimming abilities. When normality assumptions were violated, the non-parametric Kruskal–Wallis test was used. The normality of data distribution was verified using the Shapiro–Wilk test (*p* > 0.05). Post hoc multiple comparisons were conducted using the least significant difference (LSD) test for homogeneous variances (Levene’s test, *p* > 0.05) or Tamhane’s T2 test for heterogeneous variances.

### 2.4. Ethical Approval

The animal study proposal was approved by the Ethics Committee for Animal Experiments of Sichuan University (No. 2019062101). All experimental procedures were performed in accordance with the Regulations for the Administration of Affairs Concerning Experimental Animals approved by the State Council of the People’s Republic of China.

## 3. Results

### 3.1. Fish Collection and Field Environmental Conditions

A total of 142 fish were collected during the non-spawning and spawning periods. Seven fish species were used to test U_ind_ and U_burst_, while U_crit_ was tested for five fish species. The sample size for each swimming ability test ranged from 3 to 12 fish, and each fish was used only once (Table 1). Natural river water was used in keeping tanks, with a T of 20.3 °C and a DO of 6.5 mg/L during the non-spawning period, and a T of 20.4 °C and a DO of 7.12 mg/L during the spawning period. The swimming speeds of endemic fish in the Qingshui River basin were tested during the non-spawning period (October 2020) and the spawning period of the following year (April 2021). River water with 24 h of aeration, which had a DO over 7.0 mg/L, was used as the test water in this study. The temperature of the river water ranged from 17.2 to 22.4 °C during the non-spawning period and from 18.65 to 25.7 °C during the spawning period.

### 3.2. Fish Morphological Characteristics

**Figure 3:**
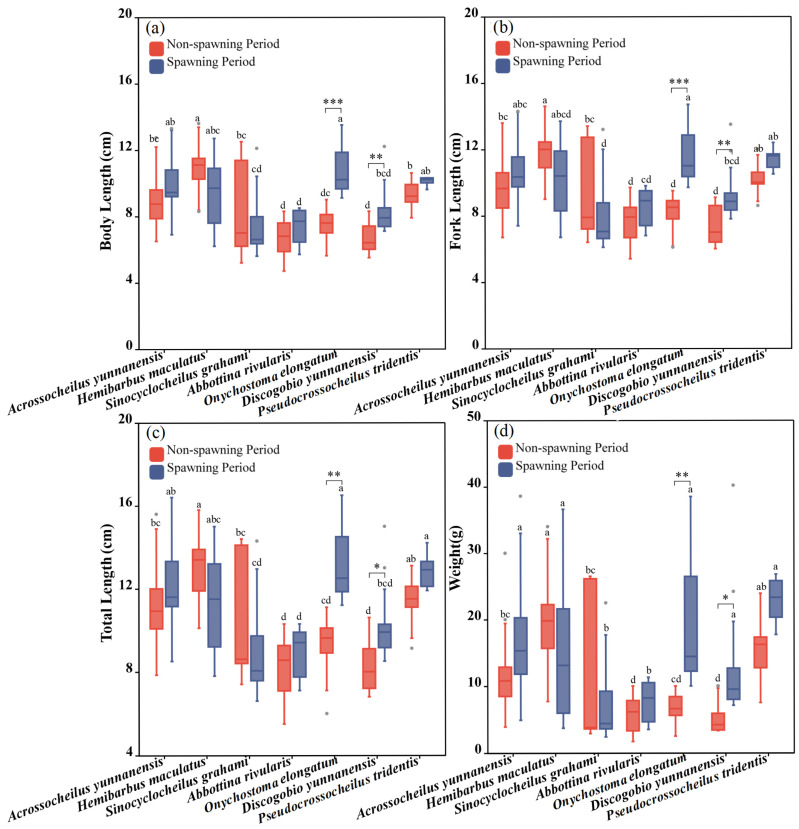
Morphological characteristics of experimental fish during the non-spawning and spawning period. (**a**–**d**) represent differences of standard body length (SL), fork length (FL), total length (TL) and weight (W) between two periods and seven studied fish, respectively. The boxes in the figure illustrate the range of lower and upper quartiles. The whiskers indicate the 1.5 interquartile range. The median values are represented by solid lines, respectively. The gray dots outside the boxes represent data that are outliers. The asterisks indicate significant differences in morphological parameters between the two periods (* indicates *p* < 0.05, ** indicates *p* < 0.01, *** indicates *p* < 0.001). Letters above boxes represents the results of post-hoc multiple comparisons.

**Figure 4:**
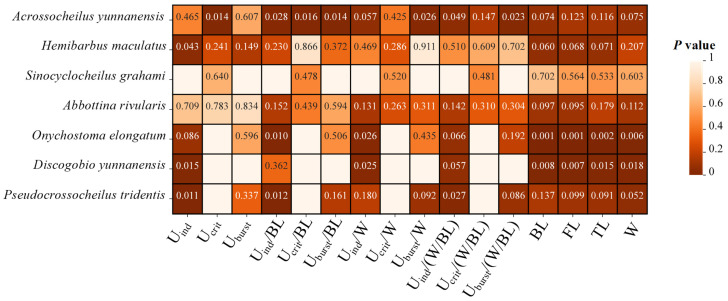
Heat map describing the differences in the swimming ability (U_ind_, U_ind_/SL, U_ind_/W, U_ind_/(W/SL), U_crit_, U_crit_/SL, U_crit_/W, U_crit_/(W/SL), U_burst_, U_burst_/SL, U_burst_/W, U_burst_/(W/SL)) of seven fish species in the Qingshui River basin between the two periods. The color in cells ranging from dark to light with P values represents the significance level from high to low, while empty cells without values indicate that the data were not obtained.

As shown in Figure 3, the SL, FL, TL and W of the seven experimental fish species differed significantly during the non-spawning period. The SL, FL, TL and W of *Hemibarbus maculatus* were significantly higher than those of other fish during the non-spawning period, with *Discogobio yunnanensis* and *Abbottina rivularis* being the lowest (one-way ANOVA for the non-spawning period: *F*_SL_ = 11.671, *p*_SL_ < 0.001; *F*_FL_ = 10.450, *p*_FL_ < 0.001; *F*_TL_ = 10.737, *p*_TL_ < 0.001; *F*_W_ = 8.665, *p*_W_ < 0.001). During the spawning period, *Onychostoma elongatum* had the largest morphological parameters among all species, while *Discogobio yunnanensis* and *Abbottina rivularis* had the smallest (one-way ANOVA for the spawning period: *F*_SL_ = 4.963, *p*_SL_ = 0.211 > 0.05; *F*_FL_ = 3.880, *p*_FL_ < 0.05; *F*_TL_ = 5.970, *p*_TL_ < 0.001; *F*_W_ = 3.408, *p*_W_ < 0.001). The morphological parameters of *Discogobio yunnanensis* and *Onychostoma elongatum* were significantly higher during the spawning period than those during the non-spawning period (independent sample T test for *Discogobio yunnanensis*: *F*_SL_ = −2.71, *p*_SL_ = 0.006, *F*_FL_ = −2.13, *p*_FL_ = 0.005, *F*_TL_ = −2.84, *p*_TL_ = 0.015, *F*_W_ = -5.86, *p*_W_ = 0.018; independent sample T test for *Onychostoma elongatum*: *F*_SL_ = −2.69, *p*_SL_ < 0.001, *F*_FL_ = −2.48, *p*_FL_ < 0.001, *F*_TL_ = −2.65, *p*_TL_ = 0.005, *F*_W_ = −4.89, *p*_W_ = 0.006), and the rest of fish species had no significant differences (Figure 4).

### 3.3. Relationship Between Swimming Ability and Fish Morphology

**Figure 5:**
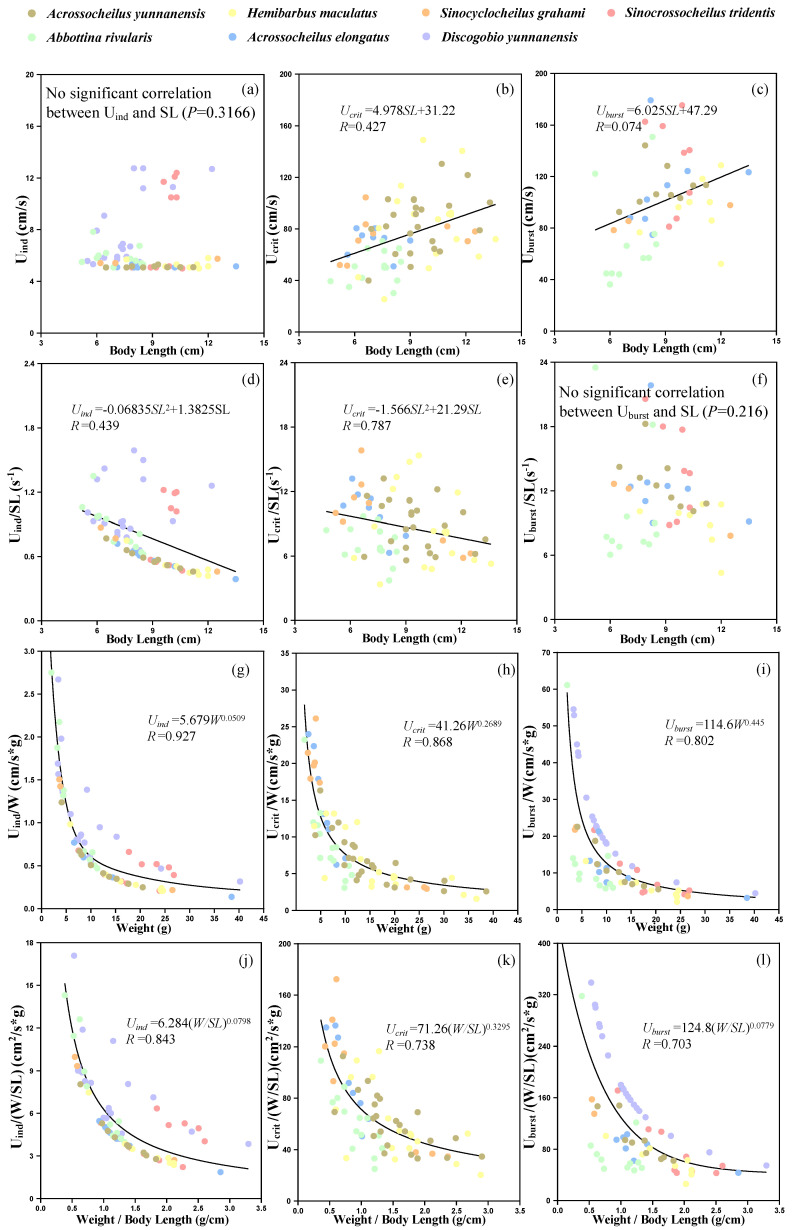
Relationship between morphological parameters and swimming ability. (**a**–**c**) represents relationship between standard body length and swimming ability (U_ind_, U_crit_ and U_burst_), respectively. (**d**–**f**) represents relationship between standard body length and swimming ability (U_ind_/SL, U_crit_/SL and U_burst_/SL), respectively. (**g**–**i**) represents relationship between weight and swimming ability (U_ind_/W, U_crit_/W and U_burst_/W), respectively. (**j**–**l**) represents relationship between weight/standard body length and swimming ability (U_ind_/(W/SL), U_crit_/(W/SL) and U_burst_/(W/SL)), respectively.

The ranges of U_ind_, U_crit_ and U_burst_ for experimental fish in the Qingshui River basin were 5–12.75 cm/s, 25.5–149 cm/s and 36.3–179.2 cm/s, respectively. Bivariate correlation shows that there was no significant correlation between U_ind_ and SL (*p* = 0.3166, Figure 5a), while U_crit_ was positively correlated with SL (*p* = 0.0002, Figure 5b). Although U_burst_ had a significant correlation with SL (*p* = 0.0227), there only existed a weak linear relationship between them (*R* = 0.074). U_ind_ and U_crit_ were negatively correlated with SL^2^ (U_ind_: *p* = 0.0001, U_crit_: *p* = 0.0204; Figure 5d,e), while U_burst_ was not found to be significantly related to SL^2^ (*p* = 0.216, Figure 5f). Compared to SL and SL^2^, fish swimming speeds showed better power function correlations with W and W/SL, where the *R* values were over 0.7 (Figure 5g–l).

### 3.4. Comparison of Swimming Ability Considering Different Periods and Species

**Figure 6:**
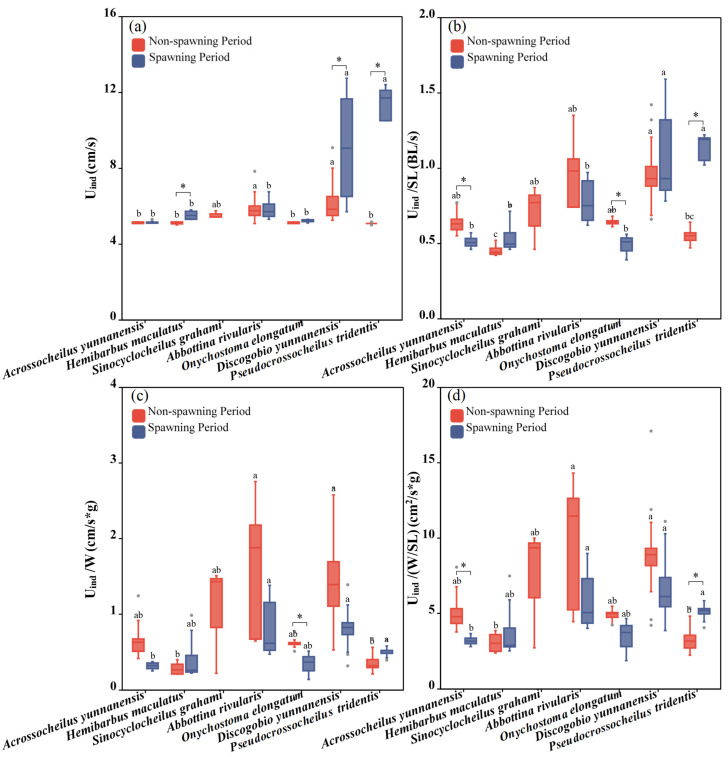
Differences between the U_ind_, U_ind_/SL, U_ind_/W and U_ind_/(W/SL) of experimental fish across species and periods. (**a**–**d**) represent differences of U_ind_, U_ind_/SL, U_ind_/W and U_ind_/(W/SL) between two periods and seven studied fish, respectively. The boxes in the figure illustrate the range of lower and upper quartiles. The whiskers indicate the 1.5 interquartile range. The median values are represented by solid lines, respectively. The gray dots outside the boxes represent data that are outliers. * represents a statistically significant difference in U_ind_ between the non-spawning and spawning periods (* indicates *p* < 0.05). Letters above boxes represents the results of post-hoc multiple comparisons.

There were significant differences in U_ind_ among the seven fish species during both the non-spawning and spawning periods (one-way ANOVA for the non-spawning period: *F* = 3.458, *p* = 0.038; one-way ANOVA for the spawning period: *F* = 44.002, *p* < 0.001; Figure 6a). *Discogobio yunnanensis* and *Abbottina rivularis* had the largest U_ind_ during the non-spawning period, while the greatest U_ind_ during the spawning period was recorded by *Pseudocrossocheilus tridentis* and *Discogobio yunnanensis*. For the same species, *Pseudocrossocheilus tridentis* (independent sample T test: *F* = −15.888, *p* = 0.011), *Discogobio yunnanensis* (independent sample T test: *F* = −2.879, *p* = 0.015) and *Hemibarbus maculatus* (independent sample T test: *F* = −3.103, *p* = 0.043) had significantly higher U_ind_ during the spawning period than those during the non-spawning period, while the rest of the fish species had no significant differences (Figure 6a).

Significant differences among fish species in U_ind_/SL, U_ind_/W and U_ind_/(W/SL) were found during both the non-spawning period and spawning period (one-way ANOVA for the non-spawning period: *F*_Uind/SL_ = 10.414, *p*_Uind/SL_ = 0.001, *F*_Uind/W_ = 7.672, *p*_Uind/W_ = 0.002, *F*_Uind/(W/SL)_ = 5.678, *p*_Uind/(W/SL)_ = 0.008; one-way ANOVA for the spawning period: *F*_Uind/SL_ = 33.775, *p*_Uind/SL_ < 0.001, *F*_Uind/W_ = 7.615, *p*_Uind/W_ = 0.004, *F*_Uind/(W/SL)_ = 8.284, *p*_Uind/(W/SL)_ = 0.003; Figure 6a–c). The significances in U_ind_/W and U_ind_/(W/SL) had the same distribution among the seven fish species during both the non-spawning period and spawning period. During the non-spawning period, *Discogobio yunnanensis* showed a stronger performance in terms of U_ind_/SL, U_ind_/W and U_ind_/(W/SL), while *Pseudocrossocheilus tridentis* and *Hemibarbus maculatus* were weaker swimmers. During the spawning period, *Discogobio yunnanensis* and *Pseudocrossocheilus tridentis* had the strongest U_ind_/SL, U_ind_/W and U_ind_/(W/SL), and *Abbottina rivularis* performed weaker in U_ind_/SL while was the strongest in U_ind_/W and U_ind_/(W/SL).

For the same species, in terms of U_ind_/SL, *Acrossocheilus yunnanensis* (independent sample T test: *F* = 2.756, *p* = 0.028) and *Onychostoma elongatum* (independent sample T test: *F* = 3.671, *p* = 0.01) swam faster during the non-spawning period than those during the spawning period, whereas *Pseudocrossocheilus tridentis* exhibited an opposite pattern (independent sample T test: *F* = −11.637, *p* = 0.012). There was no significant difference in U_ind_/SL between the two periods in the rest of the fish species (Figure 4 and Figure 6b). For the same species, considering U_ind_/W, only *Onychostoma elongatum* performed better during the non-spawning period than that during the spawning period (independent sample T test: *F* = 2.936, *p* = 0.026; Figure 4 and Figure 6c). For the same species, in terms of U_ind_/(W/SL), *Acrossocheilus yunnanensis* had a better swimming ability during the non-spawning period than that during the spawning period (independent sample T test: *F* = 2.384, *p* = 0.049), whereas *Pseudocrossocheilus tridentis* presented an opposite pattern (independent sample T test: *F* = −2.71, *p* = 0.027). There was no significant difference in U_ind_/(W/SL) between the two periods in the rest of the fish species (Figure 4 and Figure 6d).

**Figure 7:**
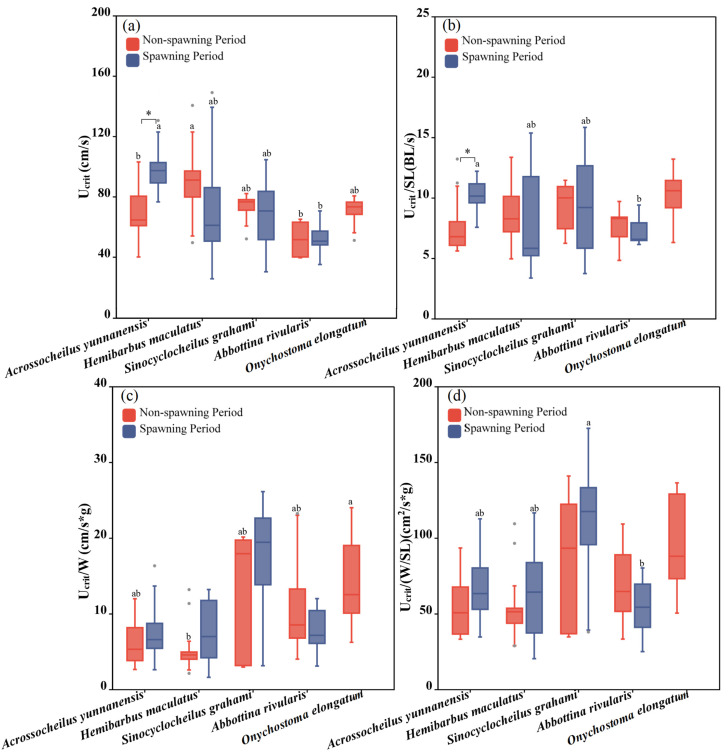
Differences between the U_crit_, U_crit_/SL, U_crit_/W and U_crit_/(W/SL) of experimental fish across species and periods. (**a**–**d**) represent differences of U_crit_, U_crit_/SL, U_crit_/W and U_crit_/(W/SL) between two periods and seven studied fish, respectively. The boxes in the figure illustrate the range of lower and upper quartiles. The whiskers indicate the 1.5 interquartile range. The median values are represented by solid lines, respectively. The gray dots outside the boxes represent data that are outliers. * represents a statistically significant difference in U_crit_ between the non-spawning and spawning periods (* indicates *p* < 0.05). Letters above boxes represents the results of post-hoc multiple comparisons (LSD or Tamhane’s T2 test, *p* < 0.05). Groups sharing no common letters differ significantly (“a” represents the highest mean value of this group, and “b” represents the lowest.), and there was no statistical difference among the groups with any of the same letters (such as “ab” and “a”, “b” and “ab”, etc.).

Five fish species were adopted to test U_crit_ during the non-spawning period (*Abbottina rivularis*, *Acrossocheilus yunnanensis*, *Onychostoma elongatum*, *Sinocyclocheilus grahami* and *Hemibarbus maculatus*), and four fish species were adopted to test the U_crit_ during the spawning period (*Abbottina rivularis*, *Acrossocheilus yunnanensis*, *Sinocyclocheilus grahami* and *Hemibarbus maculatus*). There was a significant difference among the fish species between the two periods (one-way ANOVA for the non-spawning period: *F* = 4.841, *p* = 0.003; one-way ANOVA for the spawning period: *F* = 17.344, *p* = 0.001; Figure 7a) *Hemibarbus maculatus* had the greatest U_crit_ during the non-spawning period, while *Acrossocheilus yunnanensis* exhibited the strongest performance in U_crit_ during the spawning period. The U_crit_ of fish did not exhibit a significant difference between the two periods, except for that *Acrossocheilus yunnanensis* swam faster during the non-spawning period (independent sample T test: *F* = −4.051, *p* = 0.014; Figure 4 and Figure 7a).

The significant difference in U_crit_/W among fish species was found during the non-spawning period (one-way ANOVA: *F*_Ucrit/W_ = 3.371, *p*_Ucrit/W_ = 0.045; Figure 7c), and *Onychostoma elongatum* had the highest U_crit_/W. There existed significant differences among the fish species during the spawning period in terms of U_crit_/SL and U_crit_/(W/SL) (one-way ANOVA: *F*_Ucrit/SL_ = 7.541, *p*_Ucrit/SL_ = 0.008; *F*_Ucrit/(W/SL)_ = 3.491, *p*_Ucrit/(W/BL)_ = 0.029; Figure 7b,d). *Acrossocheilus yunnanensis* swam faster in terms of U_crit_/SL than other species and *Sinocyclocheilus grahami* exhibited the strongest U_crit_/(W/SL). For the same species, in terms of U_crit_/SL, *Acrossocheilus yunnanensis* performed better during the spawning period (independent sample T test: *F* = −3.077, *p* = 0.016; Figure 4 and Figure 7b), while there was no significant difference in U_crit_/W and U_crit_/(W/SL) between the two periods for all species (Figure 4 and Figure 7c,d).

**Figure 8:**
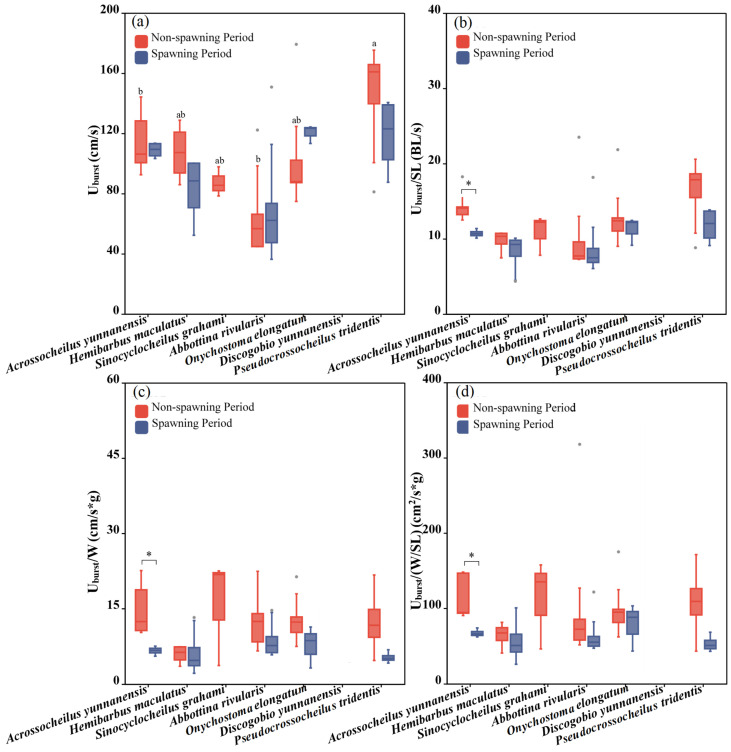
Differences between the U_burst_, U_burst_/SL, U_burst_/W and U_burst_/(W/SL) of the experimental fish across species and periods. The boxes in the figure illustrate the range of lower and upper quartiles. (**a**–**d**) represent differences of U_burst_, U_burst_/SL, U_burst_/W and U_burst_/(W/SL) between two periods and seven studied fish, respectively. The whiskers indicate the 1.5 interquartile range. The median values are represented by solid lines, respectively. The gray dots outside the boxes represent data that are outliers. * represents a statistically significant difference in U_burst_ between the non-spawning and spawning periods (* indicates *p* < 0.05). Letters above boxes represents the results of post-hoc multiple comparisons (LSD or Tamhane’s T2 test, *p* < 0.05). Groups sharing no common letters differ significantly (“a” represents the highest mean value of this group, and “b” represents the lowest.), and there was no statistical difference among the groups with any of the same letters (such as “ab” and “a”, “b” and “ab”, etc.).

Six fish species except for *Discogobio yunnanensis* were adopted to test the U_burst_ during different periods and a significant difference among fish species was found during the non-spawning period (one-way ANOVA: *F* = 3.001, *p* = 0.035) but not during the spawning period (one-way ANOVA: *F* = 2.849, *p* = 0.059). During the non-spawning period, *Acrossocheilus yunnanensis* and *Abbottina rivularis* had the smallest U_burst_ compared to the other three species (Figure 8a). For the same species, between the two periods, we did not find a significant difference in terms of U_burst_ (Figure 4 and Figure 8a).

There existed no significant difference among the fish species during both periods in terms of U_burst_/SL, U_burst_/W and U_burst_/(W/SL) (one-way ANOVA for the non-spawning period: *F*_Uburst/SL_ = 1.23, *p*_Uburst/SL_ = 0.332, *F*_Uburst/W_ = 3.348, *p*_Uburst/W_ = 0.063, *F*_Uburst/(W/SL)_ = 0.412, *p*_Uburst/(W/SL)_ = 0.835; one-way ANOVA for the spawning period: *F*_Uburst/SL_ = 0.963, *p*_Uburst/SL_ = 0.454, *F*_Uburst/W_ = 0.761, *p*_Uburst/W_ = 0.566, *F*_Uburst/(W/SL)_ = 0.562, *p*_Uburst/(W/SL)_ = 0.693). For the same species, between the two periods in terms of U_burst_/SL (independent sample T test: *F* = 3.249, *p* = 0.014), U_burst_/W (independent sample T test: *F* = 3.349, *p* = 0.026) and U_burst_/(W/SL) (independent sample T test: *F* = 3.483, *p* = 0.023), only *Acrossocheilus yunnanensis* showed a significant difference between the two periods, presenting a better swimming performance during the non-spawning period. We did not obtain the U_burst_ of *Discogobio yunnanensis* due to its U_burst_ exceeding the test range of the Loligo SY10800 used in this study. It is speculated that *Discogobio yunnanensis* had the strongest swimming performance in terms of U_burst_.

### 3.5. Factors Influencing Swimming Performance

**Figure 9:**
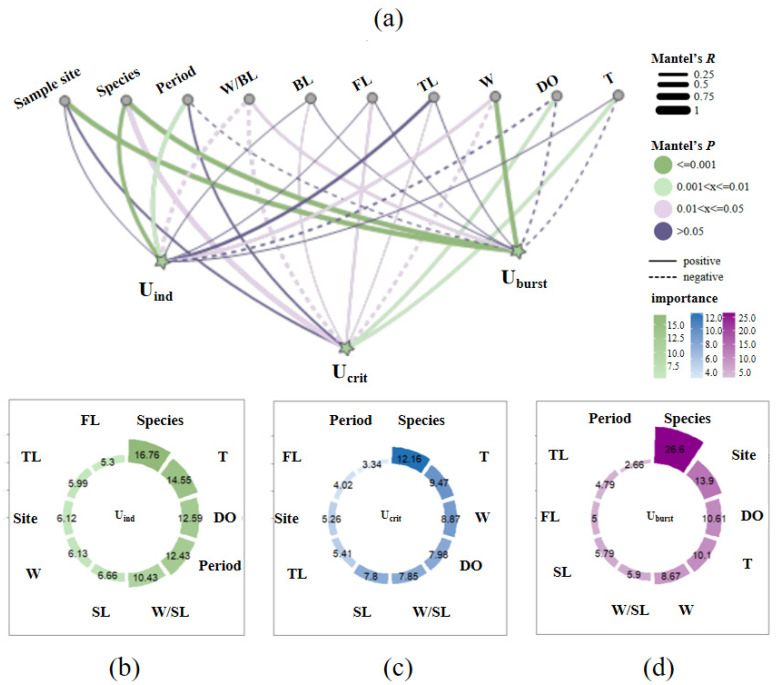
Factors influencing fish swimming ability. (**a**) depicts the correlations between fish swimming speeds and impact factors from the mantel test result, indicated by *R* and *p* values. (**b**–**d**) depict the importance ranking of impact factors for U_ind_, U_crit_ and U_burst_ from the RF result, indicated by percentage values.

As shown in Figure 9a, the result of the Euclidean distance from the mantel test indicates that weight parameters (W and W/SL) were positively related to U_crit_ (*p* < 0.05, *R* > 0) but negatively connected to U_burst_ (*p* < 0.05, *R* < 0). Weight parameters were more correlated with swimming ability than body length parameters (SL, FL and TL). The period was significantly correlated with U_ind_ (*p* = 0.002), the sample site was significantly correlated with U_burst_ (*p* = 0.001) and the DO and water temperature exhibited a significantly positive correlation with U_crit_ (for DO: *p* = 0.009, *R* = 0.195; for T: *p* = 0.008, *R* = 0.194). As shown in Figure 9b–d, the result of RF indicates that species, DO and T were the prominent factors affecting fish swimming ability in this study (% importance for species > 12.16, for DO > 7.98 and for T > 9.47). The sample site was a secondary important factor influencing U_burst_ and the period was also an important factor influencing U_ind_. Weight parameters were found to have greater effects on fish swimming speeds than the body length parameters. Generally speaking, the species identity was the main factor shaping the swimming ability, followed by environmental factors and morphological factors, combining the mantel test and RF.

## 4. Discussion

Fish swimming ability is generally associated with their morphological parameters, physiological needs and living environment [40]. A field experiment on fish swimming ability was launched to investigate the discrepancy in fish swimming speeds among different species, periods and environmental factors. Furthermore, we identified the key factors driving fish swimming ability, which could provide important technical support for the restoration of river connectivity and a reference for the conservation of rare and endemic fish in mountainous rivers.

### 4.1. The Effect of Morphological Factors on Fish Swimming Performance

In this study, the SL, FL, TL, as well as the W of fish showed a greater increase during the spawning period than during non-spawning period. This may be due to the fact that the fish are at the stage of sexual maturity and fully developed during the spawning period [41]. In this study, fish swimming ability was not strongly correlated with body length parameters (SL, FL, TL), with *r* being less than 0.6 in the fitting result, while weight parameters (W and W/SL) were found to have a better correlation with fish swimming speeds. Previous studies usually thought that body length parameters linearly correlated with fish swimming speeds [35,42,43], but our results suggest that weight-based metrics outperform length metrics in predicting swimming capacity.

Weight parameters in this study were positively related to U_crit_ but negatively connected to U_burst_. There is a complex relationship between muscle mass and drag force in fish and it varies with the fish shape and size [44,45]. For U_burst_, greater weight increased the drag force required for bursting movements and thus reduced U_burst_. Red muscle mass increases as a result of weight gain, and the larger red muscle mass allows for a more sustained release of energy available for aerobic exercise, providing sufficient fuel for U_crit_ [46,47,48]. Aerobic enhancement squeezes anaerobic expenditure, and there exists a competitive relationship between them [49]. In addition, previous studies proved that lean body mass and certain fat mass are an important physical basis for anaerobic capacity, and we also suggest introducing lean body mass and fat mass as new factors to evaluate fish U_burst_ [50,51].

### 4.2. The Effect of Species on Fish Swimming Performance

*Acrossocheilus yunnanensis* and *Onychostoma elongatum* belong to the same genus of *Acrossocheilus* and have similar habits, and their swimming capabilities were also found to be relatively close to each other. The bodies of *Acrossocheilus yunnanensis* and *Onychostoma elongatum* are slender and streamlined, causing their susceptibility to flow velocities, and consequently resulting in lower U_ind_ compared to other fish species [52]. Fish in the upper layer are more sensitive to the water current than those in the lower layer [52]. *Abbottina rivularis*, *Discogobio yunnanensis* and *Pseudocrossocheilus tridentis* have flattened thoraxes and abdomens, making them suitable for the bottom and less vulnerable to the current, and thus exhibiting greater U_ind_ [53].

Fish in the lotic environment prefer to maintain aerobic and anaerobic exercise to resist current shocks [54,55]. *Discogobio yunnanensis* and *Pseudocrossocheilus tridentis,* belonging to the same subfamily of *Labeoninae* and being active in the lotic environment, have developed physiological and metabolic characteristics as well as morphological features suitable for their environment in the process of long-term evolution and adaptation. *Discogobio yunnanensis* and *Pseudocrossocheilus tridentis* living in the lotic environment have stronger swimming abilities and have stronger resistance to the water current. The U_burst_ of *Discogobio yunnanensis* even exceeded the test range of the instrument used in this study. Instruments with bigger test ranges should be considered to obtain its U_burst_. Fish in the lentic environment typically prioritize maneuverability (U_ind_) rather than stronger swimming abilities (U_burst_ and U_crit_), often exhibiting deeper, laterally compressed bodies [9]. For example, *Abbottina rivularis* has a small body size with a big head, which is easily subject to a large current driving force, consequently resulting in a relatively weaker acceleration ability and a lower swimming ability.

### 4.3. The Effect of Life Period on Fish Swimming Performance

The U_ind_ of all fish species in this study increased to some extent during the spawning period compared to the non-spawning period, and significant differences between the two periods were found in *Hemibarbus maculatus*, *Discogobio yunnanensis* and *Pseudocrossocheilus tridentis*. We speculate that it is mainly due to the mutual adaptation between the environment and fish. During the long process of natural selection, fish species undergo self-evolution to adapt to the changes in the environment [56]. The spawning period for fish in the Qingshui River basin is from April to May, which also belongs to the rainy season of the river. During this period the amount of water in the river rises and the flow velocity increases, creating an increasing U_ind_ of fish in the basin during the spawning period [57].

The locomotion performance of fish is usually dynamic with their life history [58], and special attention should be paid to the life history effects concerning the evaluation of fish swimming ability. Changes in metabolic activities during the spawning and non-spawning period are crucial to fish swimming ability [4]. Fish swimming performance is mainly fueled by the rapid catabolism of energy storage substances (e.g., ATP, phosphocreatine, glycogen, etc.) from muscles [5]. Fish use more energy to help with reproduction during the spawning period and reduce their energy consumption for the rest of their activities [59]. In this study, the U_burst_ of fish during the spawning period was lower than during the non-spawning period. There also existed a certain degree of reduction in U_burst_/SL, U_burst_/W and U_burst_/(W/SL) during the spawning period compared to the non-spawning period for all the fish species. The reduced capacity for muscle productivity in fish was found during the spawning period due to the long-term non-use of biological muscles [60], and this functional reduction due to muscle wastage could explain the changes in U_burst_ between the two different periods in this study. However, the significant increases in body size for fish during the spawning period allow them to achieve a balance between escape and reproduction, resulting in a not-statistically significant difference in U_burst_ between the non-spawning and spawning periods.

### 4.4. Application and Future Work

This study quantified the swimming behavior of typical endemic fish species in Southwest China and analyzed the impacts of biological characteristics and environmental factors on fish swimming performance. These findings provide theoretical references and foundational data to support the conservation of rare and endemic fish resources and the restoration of connectivity in mountainous rivers in Southwest China. For instance, a growing number of fish passage facilities in impaired rivers have been constructed, aimed at restoring upstream and downstream connectivity in China [23]; however, many of them have performed ineffectively, because they were not developed for endemic fish species [61,62,63]. Data on fish swimming ability can assist in determining the appropriate hydraulic conditions for fish migration, and can subsequently help to optimize the design of fish passage facilities to guarantee the attractiveness and pass efficiency [64,65,66].

Fish swimming ability is usually related to biological characteristics and external environmental factors [40], but the driving force of fish swimming ability is currently unclear. The results of the RF test and mantel test in this study showed that the species had a prominent influence on fish swimming ability among all the factors. The species difference was the result of long-term natural evolution and profoundly affected the fish behavior. Environmental factors also had an important impact on fish swimming performance, following closely behind the species. This study evaluated the impact of two important environmental factors (DO and T) on fish swimming ability. However, fish metabolic activities are still affected by many other factors, such as photoperiod and light intensity [67,68], developmental stage [69], sex [70], population density [71], the size of the swimming ability test system [4], water flow, etc. Future works should incorporate the above factors together to construct a comprehensive prediction model of swimming ability. In addition, although abundant data on the swimming performance of endemic fish species in the Qingshui River were obtained in this study through the field experiment, some tests involved small sample sizes (e.g., n = 3 for some species–period combinations) due to logistical challenges in fish collecting. Future studies should aim for larger sample sizes to validate the findings in this study.

## 5. Conclusions

Understanding the changes in fish swimming ability and identifying the driving factors behind these changes are of pressing importance. Seven endemic fish species from the Qingshui River were taken as the objects in this study to explore the effects of biological characteristics and environmental factors on fish swimming ability. The main conclusions were as follows:

(1) Body length minimally affected U_ind_ but positively correlated with U_crit_ and U_burst_. Weight parameters (W and W/SL) showed stronger correlations with swimming performance than body length alone.

(2) Fish adapted to lotic environments (e.g., *Discogobio yunnanensis*) exhibited superior U_crit_ and U_burst_, while streamlined species displayed heightened flow sensitivity.

(3) There were significant differences in fish swimming abilities between the spawning period and non-spawning period. The U_ind_, U_ind_/SL, U_ind_/W and U_ind_/(W/SL) of *Discogobio yunnanensis* and *Pseudocrossocheilus tridentis* were higher during the spawning period, and this was similar to the U_crit_ and U_crit_/SL of *Acrossocheilus yunnanensis*. The U_burst_ of all fish species was smaller during the spawning period.

(4) The swimming ability was mainly influenced by the species identity, followed by environmental conditions and morphological factors. Species, DO and T were the prominent factors affecting the three types of swimming ability in this study. The sample site was a secondary important factor influencing U_burst_ and the period was also an important factor influencing U_ind_.

## Figures and Tables

**Table 1:** Sample sizes for swimming ability tests (U_ind_, U_crit_ and U_burst_) across seven fish species during spawning and non-spawning periods. Slashes (/) indicate that tests were not conducted.

Test Period	Fish Species	Sample Size		
		U_ind_	U_crit_	U_burst_
Non-Spawning Period	*Acrossocheilus yunnanensis*	5	11	5
	*Hemibarbus maculatus*	4	11	4
	*Sinocyclocheilus grahami*	3	5	3
	*Abbottina rivularis*	5	5	5
	*Onychostoma elongatum*	5	8	5
	*Discogobio yunnanensis*	9	/	9
	*Pseudocrossocheilus tridentis*	5	/	5
Spawning Period	*Acrossocheilus yunnanensis*	4	12	4
	*Hemibarbus maculatus*	4	7	4
	*Sinocyclocheilus grahami*	/	4	/
	*Abbottina rivularis*	6	9	6
	*Onychostoma elongatum*	3	/	3
	*Discogobio yunnanensis*	12	/	12
	*Pseudocrossocheilus tridentis*	5	/	4

## Data Availability

The original contributions presented in this study are included in the article/Supplementary Materials. Further inquiries can be directed to the corresponding author.

## Supplementary Materials

The following supporting information can be downloaded at: https://www.mdpi.com/article/10.3390/ani15121819/s1.

## Abbreviations

The following abbreviations are used in this manuscript:

U_ind_	Induced Swimming Speed
U_crit_	Critical Swimming Speed
U_burst_	Burst Swimming Speed
SL	Standard Body Length
FL	Fork Length
W	Weight
W/SL	Weight-to-Standard-Body-Length Ratio
TL	Total Length
DO	Dissolved Oxygen
T	Temperature
RF	Random Forest
QSH	Qingshuihe Hydropower Station
BSJ	Bisongjiu Hydropower Station
GLI	Gelei I Hydropower Station
GLII	Gelei II Hydropower Station
SB	Shibie Hydropower Station
HPY	Houpayan Hydropower Station
BD	Bada Hydropower Station
